# Study on diagnostic models for insomnia and gastralgia with Liver-Spleen Disharmony Syndrome based on machine learning

**DOI:** 10.3389/fmed.2026.1782578

**Published:** 2026-05-26

**Authors:** Enshi Lu, Xiaoliang Zhao, Hongjiao Li, Xuehua Sun, Liyun He

**Affiliations:** 1 Institute of Basic Research in Clinical Medicine, China Academy of Chinese Medical Sciences, Beijing, China; 2 Department of Liver Diseases, Shuguang Hospital Affiliated to Shanghai University of Traditional Chinese Medicine, Shanghai, China

**Keywords:** CHAID algorithm, diagnostic model, Liver-Spleen Disharmony Syndrome, machine learning, Traditional Chinese Medicine

## Abstract

**Background:**

The prevalence of insomnia and gastralgia has recently increased. Traditional Chinese Medicine (TCM) offers unique advantages in treating these conditions. Accurate syndrome differentiation is essential for effective treatment. However, as a common syndrome in TCM for these conditions, the diagnostic criteria for Liver-Spleen Disharmony Syndrome (LSDS) currently lack uniformity.

**Objective:**

To develop a machine learning-based diagnostic model for LSDS in patients with insomnia and/or gastralgia.

**Methods:**

In this prospective observational study, clinical data from 575 patients with insomnia and/or gastralgia were collected using structured scales. The data included demographic information and clinical symptoms related to LSDS. Six machine learning algorithms-Chi-square Automatic Interaction Detector (CHAID), C5.0 decision tree, Back-Propagation Neural Network (BPNN), Radial Basis Function (RBF) network, Bayesian Network (BN), and Binomial Logistic Regression Analysis (BLRA)-were employed to construct diagnostic models for LSDS. Model performance was evaluated using receiver operating characteristic (ROC) curves, confusion matrices, and precision-recall (P-R) curves.

**Results:**

The CHAID model outperformed all others, achieving an area under the ROC curve (AUC) of 0.889 (95% CI: 0.861–0.914), accuracy (AC) of 0.960, sensitivity (SE) of 0.972, specificity (SP) of 0.977, and an F1 score of 0.955. Prediction of variable importance in the CHAID model indicated that depression or irritability was the most important symptom variable for LSDS, followed by epigastric fullness, distending pain in hypochondrium, poor excretion of stool, diarrhea with abdominal pain, and excessive flatus. The C5.0 model ranked second (AUC = 0.797, accuracy = 0.958, F1 = 0.953). Other models showed progressively lower performance: BLRA (AUC = 0.648, accuracy = 0.947, F1 = 0.942), BPNN (AUC = 0.697, accuracy = 0.913, F1 = 0.901), BN (AUC = 0.538, accuracy = 0.944, F1 = 0.935), and RBF (AUC = 0.569, accuracy = 0.824, F1 = 0.810).

**Conclusion:**

We successfully developed a machine learning-based diagnostic model for Liver-Spleen Disharmony Syndrome (LSDS) in patients with insomnia and gastralgia. The CHAID model demonstrated superior performance and shows promise as an exploratory tool to assist clinicians; however, independent external validation is required before it can be considered for clinical application.

## 1 Introduction

Insomnia, recognized globally as a significant public health concern, imposes substantial economic and health burdens on medical and health systems (1). Over recent years, its prevalence has surged across all regions (2, 3), profoundly affecting people’s health and quality of life (4). Concurrently, stomach issues, such as acute and chronic gastritis, peptic ulcers, reflux oesophagitis, and functional dyspepsia, commonly manifest with upper abdominal pain. Studies indicate a close correlation between insomnia and stomach discomfort, mediated by bidirectional communication between the gastrointestinal tract and the brain through the microbiota–gut–brain axis (5, 6). This axis plays a vital role in regulating insomnia by modulating neuronal signals, bacterial metabolites, and immune responses (7). However, diminished sleep quality can alter intestinal microbe composition, precipitating gastrointestinal diseases (8, 9). In addition, negative emotions significantly impact both insomnia and Gastralgia, with previous studies indicating their potential as prodromal symptoms of anxiety and depression (10, 11).

Traditional Chinese medicine (TCM) has historically played a vital role in disease prevention and healthcare in China (12, 13). Insomnia and gastralgia are dominant diseases associated in TCM (14, 15). Although TCM is still regarded as a complementary and alternative therapy in modern medicine, it has attracted wide attention due to its individualized diagnostic approach, treatment characteristics, and unique therapeutic effect in the treatment of insomnia and gastralgia (16, 17). Accurate syndrome differentiation is an important prerequisite for achieving curative effects (18). Liver-Spleen Disharmony Syndrome (LSDS) is one of the most common syndromes associated with insomnia and Gastralgia. TCM posits that the liver’s regulatory function on the body’s qi is pivotal, disruptions in mood and liver qi can impede the spleen and stomach’s functions, leading to an imbalance between the liver and spleen and subsequent insomnia or stomach discomfort.

Machine learning (ML), as one of the core algorithms of artificial intelligence, has found widespread application in various medical fields, including disease diagnosis, personalized treatment, clinical decision support, and drug development (19). By analyzing patient medical data, imaging, and genetic information, ML has significantly enhanced early diagnosis capabilities and optimized treatment plans for diseases such as cancer, cardiovascular conditions, and hematological disorders (20). Moreover, ML has accelerated the drug development process, providing innovative solutions, particularly in the study of antimicrobial resistance (21). ML is also widely used in the development of clinical decision support systems and diagnostic tools. For instance, Yıldız et al. (22) employed a machine learning model based on clinical parameters to predict temporomandibular joint disorders, offering efficient and accurate clinical diagnostic support. As technology continues to advance, machine learning is poised to play an increasingly critical role in driving the development of precision medicine and the future of intelligent healthcare.

In TCM, machine learning has also demonstrated significant potential. By analyzing large volumes of symptom, sign, and case data, machine learning can help standardize and objectify the diagnosis and treatment processes in TCM, thereby improving clinical efficiency and accuracy (23–25). However, despite some exploratory studies focusing on the diagnosis of insomnia, gastralgia, and other symptoms in TCM, research on LSDS remains limited. This has resulted in a lack of unified diagnostic criteria, with significant variations in the identification and treatment of LSDS across different clinical practices.

Notably, while current diagnostic frameworks for functional gastrointestinal disorders, such as the Rome IV criteria, are primarily based on symptom patterns, the TCM concept of Liver-Spleen Disharmony Syndrome (LSDS) offers a complementary, holistic perspective. Specifically, the upper gastrointestinal symptoms of LSDS (gastralgia, epigastric fullness) closely align with functional dyspepsia; the intestinal manifestations (poor excretion of stool, diarrhea with abdominal pain) correspond to irritable bowel syndrome (26); and the emotional symptoms (depression, irritability) are strongly associated with anxiety and depressive disorders (27). The comorbidity between functional gastrointestinal disorders and mood disturbances involves multiple interconnected pathways, including the brain-gut axis, gut microbiota dysbiosis, and neuroimmune mechanisms (28, 29). Thus, LSDS can be conceptualized as an integrative diagnostic framework for identifying a patient subgroup characterized by the comorbidity of functional gastrointestinal disorders and emotional disturbances. This framework not only enhances our understanding of the heterogeneity of functional gastrointestinal disorders but also provides a novel basis for personalized treatment strategies.

To address this gap, we collected clinical data based on the symptoms and signs of LSDS, developed diagnostic models using six machine learning algorithms, and identified the optimal model to support TCM diagnosis and treatment.

## 2 Data and methods

### 2.1 Study design and population

The Ethics Committee of the Institute of Basic Research in Clinical Medicine, China Academy of Chinese Medical Sciences, approved all experimental protocols related to this study (approval ID: P22002/PJ02) and confirmed that informed consent was obtained. In this prospective multicentre observational study, our objective was to establish a diagnostic model for LSDS in cases of insomnia and gastralgia using various machine learning algorithms. This aimed to provide a convenient and efficient tool for clinical diagnosis. All data were collected from patients with insomnia and/or gastralgia at the Shijitan Hospital of Capital Medical University and the Guang’anmen Hospital of the China Academy of Chinese Medical Sciences.

**FIGURE 1:**
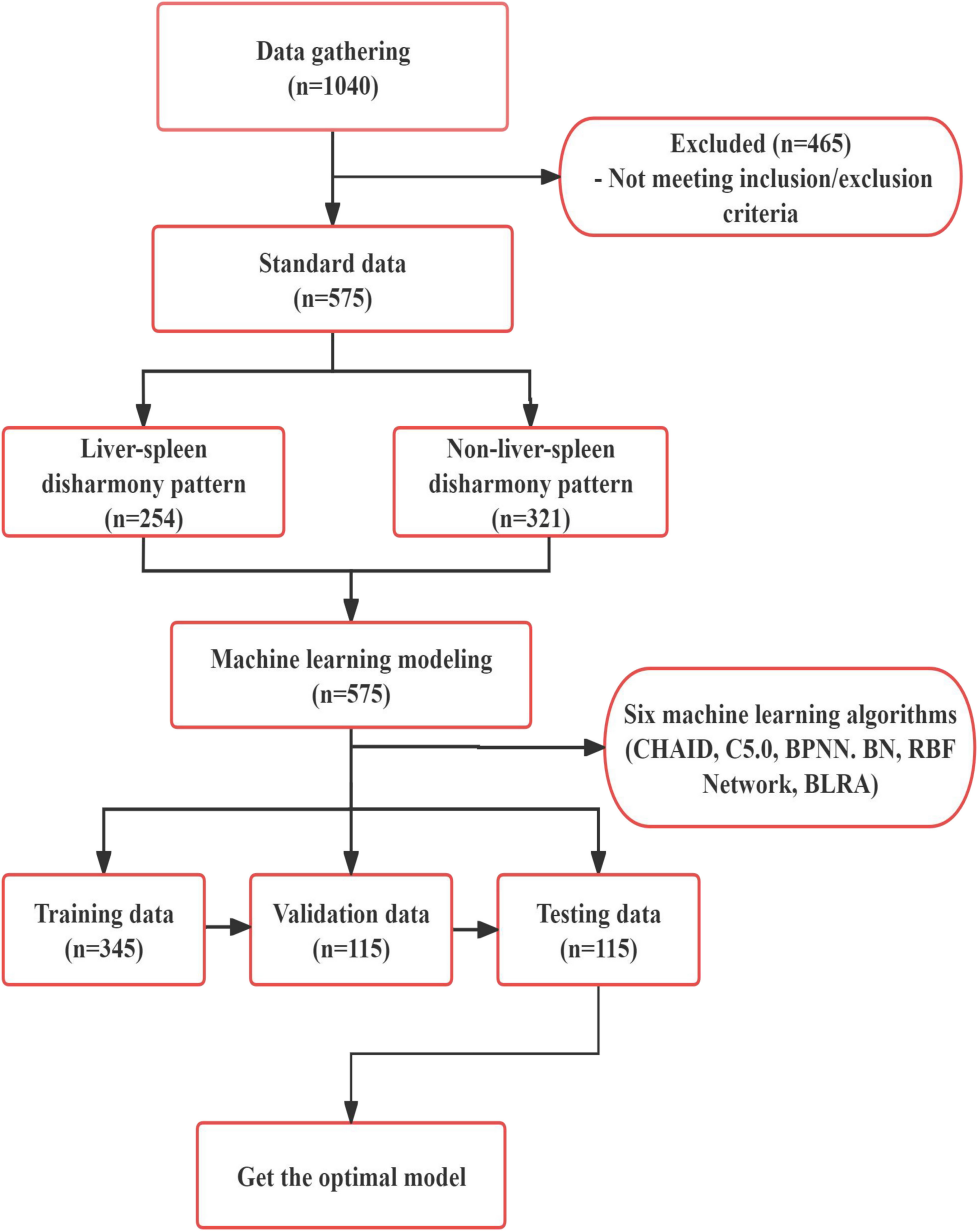
Flow chart of patient selection and model construction. CHAID, chi-square automatic interaction detector; BPNN, back-propagation neural network; RBF, radial basis function; BN, Bayesian network; BLRA, binomial logistic regression analysis.

Patients were diagnosed with insomnia and/or Gastralgia according to Chinese Guidelines for the Diagnosis and Treatment of Insomnia (2017) (30), the International Classification of Sleep Disorders (ICSD-3) (2014) and the Chinese Consensus on Chronic gastritis (2017) (31). The TCM diagnostic criteria for LSDS referred to the “Expert Consensus on the Diagnosis and Treatment of TCM Liver-Spleen Disharmony Syndrome” (2017) (32). Inclusion criteria were: (1) chief complaint of Gastralgia and/or insomnia meeting the diagnostic criteria of the above diseases; (2) age 18–65 years old; (3) mild to moderate depression and anxiety may be present. Exclusion criteria were: (1) combined with uncontrolled or serious diseases, such as malignant tumors, persistent pain, cardiovascular and cerebrovascular diseases, and endocrine diseases, etc.; (2) severe depression or psychosis, such as major depressive disorder with active suicidal ideation, schizophrenia, or bipolar disorder with manic episodes; (3) lactating patients. A total of 1,040 clinical data points were collected from September 2022 to September 2024, and 575 data points were selected as research samples after screening. A flowchart of the patient selection and model construction for this study is shown in Figure 1.

### 2.2 Collection of clinical information

According to the purpose of the study, a clinical information questionnaire for LSDS patients with insomnia and/or gastralgia was designed. The main contents included demographic data, past medical history, Pittsburgh Sleep Quality Index, Gastrointestinal Symptom Rating Scale, Hamilton Depression Scale (HAMD), Hamilton Anxiety Scale (HAMA), LSDS main symptoms grading table, TCM syndrome differentiation and treatment information. Information was collected from patients who met the inclusion and exclusion criteria, and written informed consent was obtained from the participants before collection.

The diagnosis of LSDS vs. non-LSDS was made jointly by three TCM chief physicians, each with more than 10 years of clinical experience, based on the 2017 “Expert Consensus on the Diagnosis and Treatment of TCM Liver-Spleen Disharmony Syndrome.” All physicians involved in syndrome differentiation received standardized training before the study began to ensure consistency in diagnostic criteria. For difficult or ambiguous cases, a consensus diagnosis was reached through group discussion among the three physicians.

HAMD and HAMA were examined by trained TCM practitioners using conversation and observation. After the examination, the doctors graded the patients on these scales. For example, the HAMD item 8, “block (slow thinking and speech, poor concentration, decreased initiative),” needs to be assessed based on the doctor’s observation of the patient. Item 4, “difficulty falling asleep,” was rated based on the patient’s own verbal narrative. The first item of “depression” requires both. Thus, the objectivity of the scale evaluation is guaranteed. All data collection was carried out by qualified TCM doctors, which ensured the credibility of information collection.

### 2.3 Quality Control

Before the collection of clinical information, researchers at multiple centers underwent training to understand the study’s purpose, methods, procedures, and scale collection standards. Diagnostic and exclusion criteria were strictly implemented in the trial, and unified and standardized information collection standards were adopted to minimize selection and information bias. To ensure the authenticity and reliability of the research results, a combination of internal checks and external monitoring was adopted. Additionally, aperiodic on-site or telephone monitoring of each research center was conducted to maintain the randomness and independence of the monitoring, thereby accurately assessing the implementation of the research at each center

### 2.4 Model application and performance evaluation

Diagnosing TCM syndromes can be challenging and often requires experienced TCM physicians for accurate diagnosis and treatment. Compared to deep learning, machine learning algorithms can yield better results with limited clinical data. Additionally, since the variables and results of this study were binary data, the classification algorithm under supervised learning was more effective. Therefore, six machine learning models were used: chi-square automatic interaction detection (CHAID), C5.0 decision tree (C5.0), back propagation neural network (BPNN), radial basis function network (RBF Network), Bayesian network (BN), and binomial logistic regression analysis (BLRA). The study focused on developing a diagnostic model for LSDS.

All algorithms were implemented using the default hyperparameter settings of SPSS Modeler 18.0. CHAID decision tree: maximum tree depth = 10, minimum child node size = 5, early stopping enabled; C5.0 decision tree: pruning severity = 75%, minimum cases = 2, trials = 10; BPNN (Back-Propagation Neural Network): single hidden layer (6 nodes), learning rate = 0.3, momentum = 0.2, epochs = 500; RBF network (Radial Basis Function network): spread = 1.0, number of hidden units automatically determined; Bayesian network: tree-augmented naïve Bayes (TAN) structure with maximum likelihood parameter estimation; Binomial Logistic Regression Analysis (BLRA): default settings. It should be noted that no hyperparameter grid search or manual tuning was performed in this study.

The validation strategy of this study was as follows: ① For the CHAID and C5.0 decision trees, 10-fold cross-validation was enabled in the independent modeling nodes; ② For BPNN, RBF, Bayesian network, and BLRA, a unified partition validation (training:test:validation = 6:2:2) was adopted in the Auto Classifier node. It should be noted that, due to software architecture limitations, the independent node for the Bayesian network does not support the cross-validation option.

Model performance was evaluated using simultaneous accuracy (AC), sensitivity (SE), specificity (SP), positive predictive value (PPV), negative predictive value (NPV), F1 score (F1), the area under the ROC curve (AUC), and precision-recall (P-R) curves.

AC denotes the proportion of correctly predicted samples within the overall sample. Where TP, FP, TN, and FN denote the numbers of true positives, false positives, true negatives, and false negatives, respectively. The calculation formulas are shown in Equations 1–6.


$$\text{AC}=\frac{\left(\mathrm{TP}+\mathrm{TN}\right)}{\left(\mathrm{TP}+\mathrm{FP}+\mathrm{TN}+\mathrm{FN}\right)}$$ (1)

SE reflects the proportion of positive patients identified as positive, with no recall indicating a missed patient. The formula used is as follows:


$$S\,E=\frac{T\,P}{\left(T\,P+F\,N\right)}$$ (2)

SP refers to the proportion of negative patients who are identified as negative, and no recall indicates misdiagnosis. The formula used is as follows:


$$S\,P=\frac{T\,N}{\left(T\,N+F\,P\right)}$$ (3)

PPV refers to the proportion of people who are truly positive in the test. The formula used is as follows:


$$P\,P\,V=\frac{T\,P}{\left(T\,P+F\,P\right)}$$ (4)

NPV indicates the proportion of the population with a negative test result that is indeed negative and can be calculated as follows:


$$N\,P\,V=\frac{T\,N}{\left(T\,N+F\,N\right)}$$ (5)

F1 refers to the comprehensive index of precision and recall, in which precision (P) is equivalent to PPV and recall (R) is equivalent to SE. The calculation formula is as follows.


$$F\,1=\frac{2\,P\,R}{\left(P+R\right)}$$ (6)

### 2.5 Statistical analysis

Statistical descriptions and analyses were conducted using SPSS software (version 26.0). A *t*-test or non-parametric test was used for measurement data, and the chi-square test was used for technical data. Statistical significance was set at *P* < 0.05. Utilizing statistically significant TCM symptoms as independent variables and “LSDS” as dependent variables, SPSS Modeler18.0 software was used to construct six diagnostic models. The data were randomly partitioned into training, test, and validation sets at a ratio of 6:2:2 using the Partition node. The optimal diagnostic model was identified through repeated verification, and its performance was evaluated on the validation set using the confusion matrix, P-R curve, ROC curve, AUC, accuracy, sensitivity, specificity, positive predictive value, negative predictive value, and F1 score. Notably, AUC and F1-score served as the primary evaluation metrics, with accuracy as a secondary metric, and the precision-recall curve was also included.

The dataset contained a small proportion of missing values, with each variable having a missing rate below 5% and an approximately random distribution. Continuous variables were imputed using the median, while missing values for categorical variables were treated as a separate category to preserve all available samples. After this processing, 575 cases were included in the final analysis.

## 3 Results

### 3.1 No significant difference in demographics between LSDS and NLSDS patients

Overall, 1040 clinical cases were initially included in this study, and after screening, 575 cases remained. Table 1 shows the demographic differences between patients with LSDS and NLSDS. Among them, 254 patients had LSDS, comprising 161 females (63.4%) with an average age of 45.7 years. Additionally, 321 patients were diagnosed with non-LSDS (NLSDS). No significant differences in demographic factors such as age or sex were observed between the two groups (*P* ≥ 0.05).

**TABLE 1:** Comparison of demographic data of patients.

Characteristic	LSDS (*n* = 254)	Non-LSDS (*n* = 321)	*P*-value
Sample size, n (%)	254 (44.2)	321 (55.8)	
Sex, n (%)			0.732
Male	93 (36.6)	122 (38.0)	
Female	161 (63.4)	199 (62.0)	
Age, years	45.7 ± 11.5	45.6 ± 11.6	0.980
BMI, kg/m^2^	23.3 ± 4.1	23.1 ± 3.9	0.558

### 3.2 Significant differences in TCM symptoms between LSDS and NLSDS patients

Analysis of clinical symptoms revealed 27 items with notable differences between the 2 groups, as summarized in Table 2. Compared with NLSDS patients, LSDS patients were more likely to have epigastric fullness, depression, irritability, fatigue, poor stool excretion, frequent sighing, bitter taste in the mouth, anxiety, Gastralgia, sour regurgitation, hiccups, loose stools, increased flatulence, distending pain in hypochondrium, and diarrhea with abdominal pain, among other symptoms. However, symptoms such as frequent waking at night or early awakening are more common in patients with NLSDS. These differences were statistically significant (*P* < 0.05).

**TABLE 2:** Comparison of TCM symptoms between LSDS and NLSDS.

	TCM symptoms	LSDS (*n* = 254)	NLSDS (*n* = 321)	*P*-value
1	Epigastric fullness	218 (85.2)	48 (14.9)	<0.001
2	Depression or irritability	212 (83.4)	128 (39.8)	<0.001
3	Fatigue	197 (77.5)	163 (50.7)	<0.001
4	Poor excretion of stool	187 (73.6)	53 (16.5)	<0.001
5	Frequent waking at night or early awakening	187 (73.6)	203 (63.2)	0.008
6	Frequent sighing	146 (57.4)	103 (32.1)	<0.001
7	Bitter taste in the mouth	140 (55.1)	113 (35.2)	<0.001
8	Anxiety	134 (52.7)	99 (30.8)	<0.001
9	Gastralgia	123 (48.4)	78 (24.3)	<0.001
10	Sour regurgitation	118 (46.4)	104 (32.3)	0.001
11	Hiccup	112 (44.1)	101 (31.4)	0.002
12	Loose stool	108 (42.5)	91 (28.3)	<0.001
13	Excessive flatus	101 (39.7)	83 (25.8)	<0.001
14	Dry mouth	101 (39.7)	89 (27.7)	0.002
15	Superficial sleep	95 (37.4)	90 (28.1)	0.017
16	Distending pain in hypochondrium	93 (36.6)	18 (5.6)	<0.001
17	Heartburn	91 (35.8)	81 (25.2)	0.006
18	Dreaminess	91 (35.8)	81 (25.2)	0.006
19	Sensation of chill	85 (33.4)	48 (14.9)	<0.001
20	Depression	79 (31.1)	45 (14.1)	<0.001
21	Diarrhea with abdominal pain	75 (29.5)	19 (5.9)	<0.001
22	Hyperactive bowel sounds	75 (29.5)	53 (16.5)	<0.001
23	Increased frequency of bowel movements	73 (28.7)	43 (13.4)	<0.001
24	Difficulty falling asleep after waking	72 (28.3)	57 (17.7)	0.003
25	Palpitation	67 (26.4)	44 (13.7)	<0.001
26	Abdominal hunger pain	65 (25.6)	38 (11.8)	<0.001
27	Nausea and vomiting	65 (25.5)	44 (13.7)	<0.001

### 3.3 Overall optimal performance of CHAID model among six machine-learning algorithms

Utilizing the 27 TCM symptoms with significant differences as independent variables, diagnostic models were constructed based on six machine learning algorithms. The overall performance comparison of the six machine learning algorithms is presented in Table 3. Compared with that of the other algorithms, the overall performance of the diagnostic model constructed using CHAID was better, and its AUC (0.889, 95%CI 0.861–0.914), accuracy, sensitivity, specificity, positive predictive value, F1 score, and negative predictive value were better compared to other models.

**TABLE 3:** Performance evaluation of diagnostic models for six machine learning algorithms.

Model	AC	SE	SP	PPV	NPV	F1	AUC and 95%CI
CHAID	0.960	0.972	0.977	0.939	0.977	0.955	0.889(0.861, 0.914)*
C5.0	0.958	0.972	0.957	0.935	0.977	0.953	0.797(0.762, 0.829)
BPNN	0.913	0.905	0.919	0.898	0.919	0.901	0.697(0.658, 0.735)
BLRA	0.947	0.972	0.928	0.914	0.977	0.942	0.648(0.607,0.687)
RBF Network	0.824	0.850	0.803	0.774	0.871	0.810	0.569(0.528,0.610)
BN	0.944	0.921	0.962	0.951	0.939	0.935	0.538(0.497,0.580)

AC, accuracy; SE, sensitivity; SP, specificity; PPV, positive predictive value; NPV, negative predictive value; F1, F1 score; AUC, area under the curve.

*The CHAID model demonstrated the best overall performance.

**FIGURE 2:**
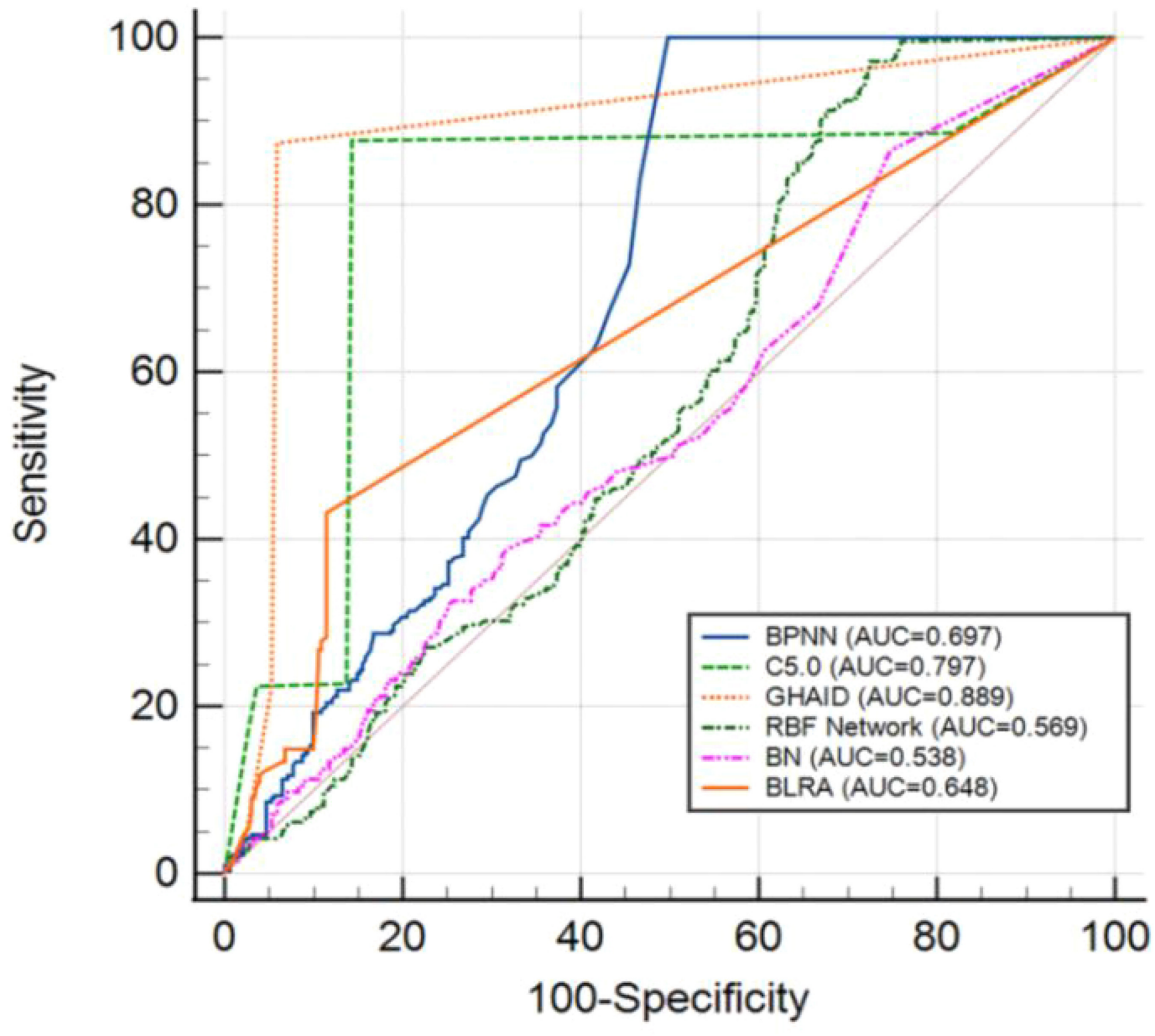
Evaluation of the six machine-learning algorithms based on the AUC of the ROC curve. ROC, receiver operating characteristic. The horizontal coordinate (X axis) represents specificity, or the false-positive rate, and the vertical coordinate (Y axis) represents sensitivity, or the true-positive rate. The closer the curve is to the top left corner (smaller X, larger Y), the higher the prediction accuracy. AUCs of six different models (represented by lines of different colors) are shown in the legend. The higher the AUC, the higher the prediction accuracy.

**FIGURE 3:**
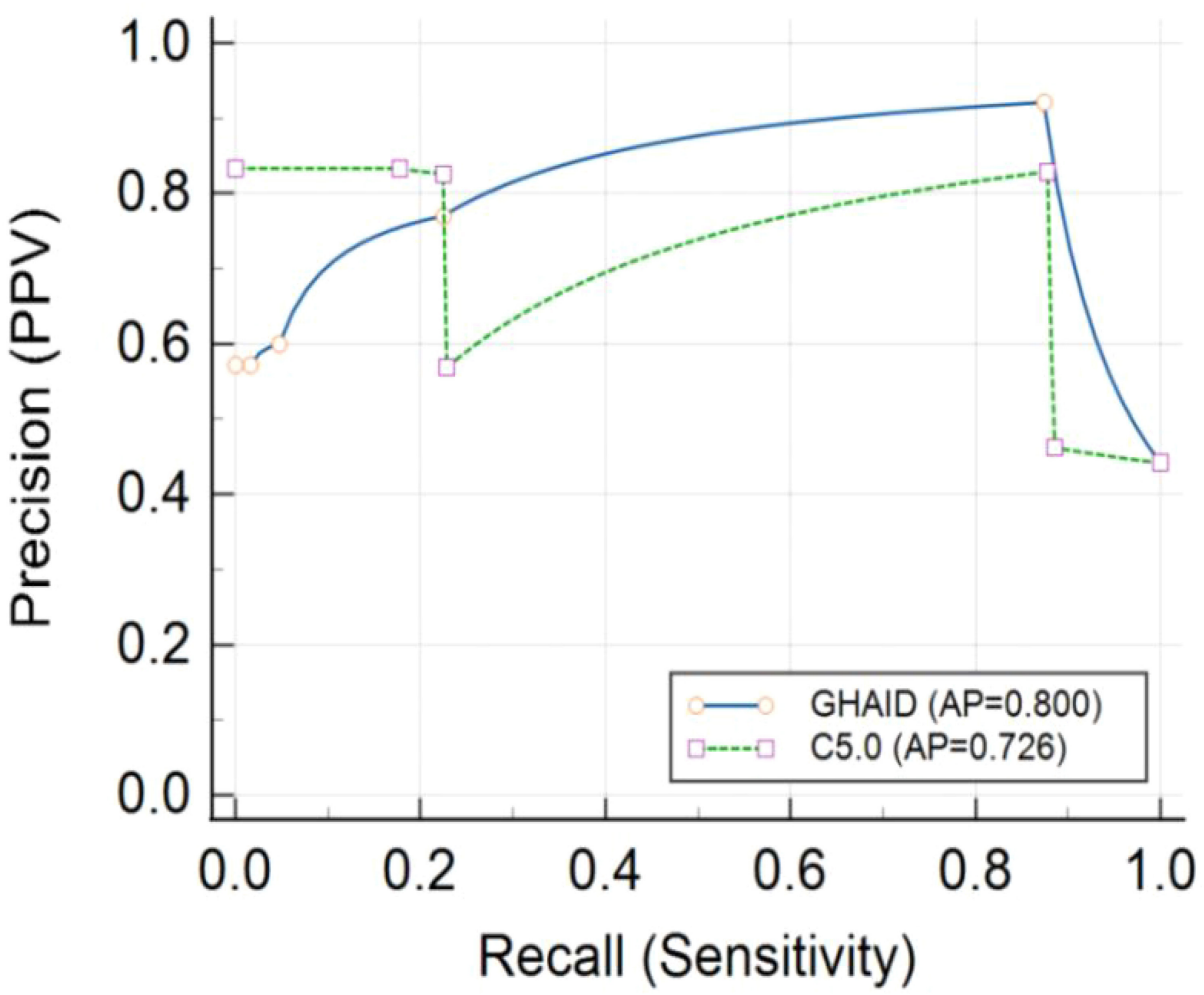
P-R curves for the six machine-learning algorithms. Horizontal coordinates represent recall, and vertical coordinates represent precision. The closer the P-R curve is to the upper right corner (precision and recall are close to 1), the better the model better. P-R curve: accurate recall curve; AP: average accuracy.

**FIGURE 4:**
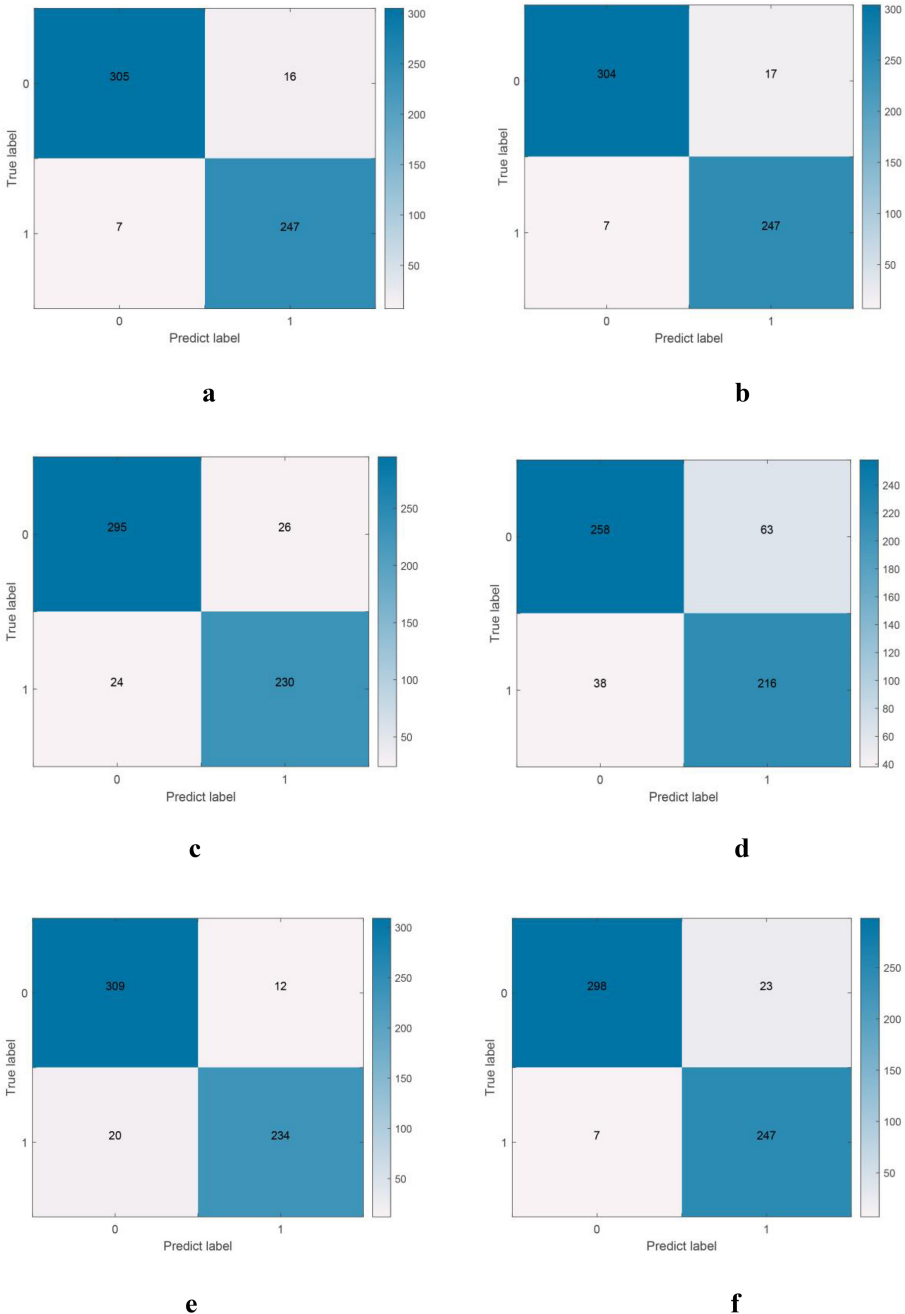
Confusion matrix. **(a)** CHAID, **(b)** C5.0, **(c)** BPNN, **(d)** RBF Network, **(e)** BN, **(f)** BLRA. In the figure, “1” represents patients with LSDS and “0” represents those with NLSDS. The vertical coordinates represent the actual TCM syndrome type, and the horizontal coordinates represent the predicted value made by the machine learning algorithm. The blue area represents the number of cases in which the predicted value matched the actual value, and the white area represents the number of cases in which the predicted value did not match the actual value.

Figure 2 show the AUC of ROC curves of six machine-learning models. The larger the AUC, the better the classification effect of the model. The AUC of the CHAID model (0.889) was the highest, followed by that of the C5.0 model (0.787). This indicates that the CHAID model and C5.0 model have high diagnostic value for LSDS patients. On this basis, the P-R curves of CHAID and C5.0 models were drawn, and the average accuracy (AP) of the two models was calculated. The results show that the AP of the CHAID model (0.800) is better than that of the C5.0 model (0.726) (Figure 3). Figure 4 shows the confusion matrix diagram of the six machine-learning models. In this study, 575 cases were included, including 254 LSDS cases and 321 NLSDS cases. Figure 4a shows that the number of cases accurately predicted by the CHAID model for LSDS and NLSDS were 247 and 305 cases, respectively. The total number of cases accurately predicted was 552 cases, and the prediction accuracy was 0.96, which was better than that of the other 5 models.

### 3.4 CHAID diagnostic model analysis and variable interpretation

**FIGURE 5:**
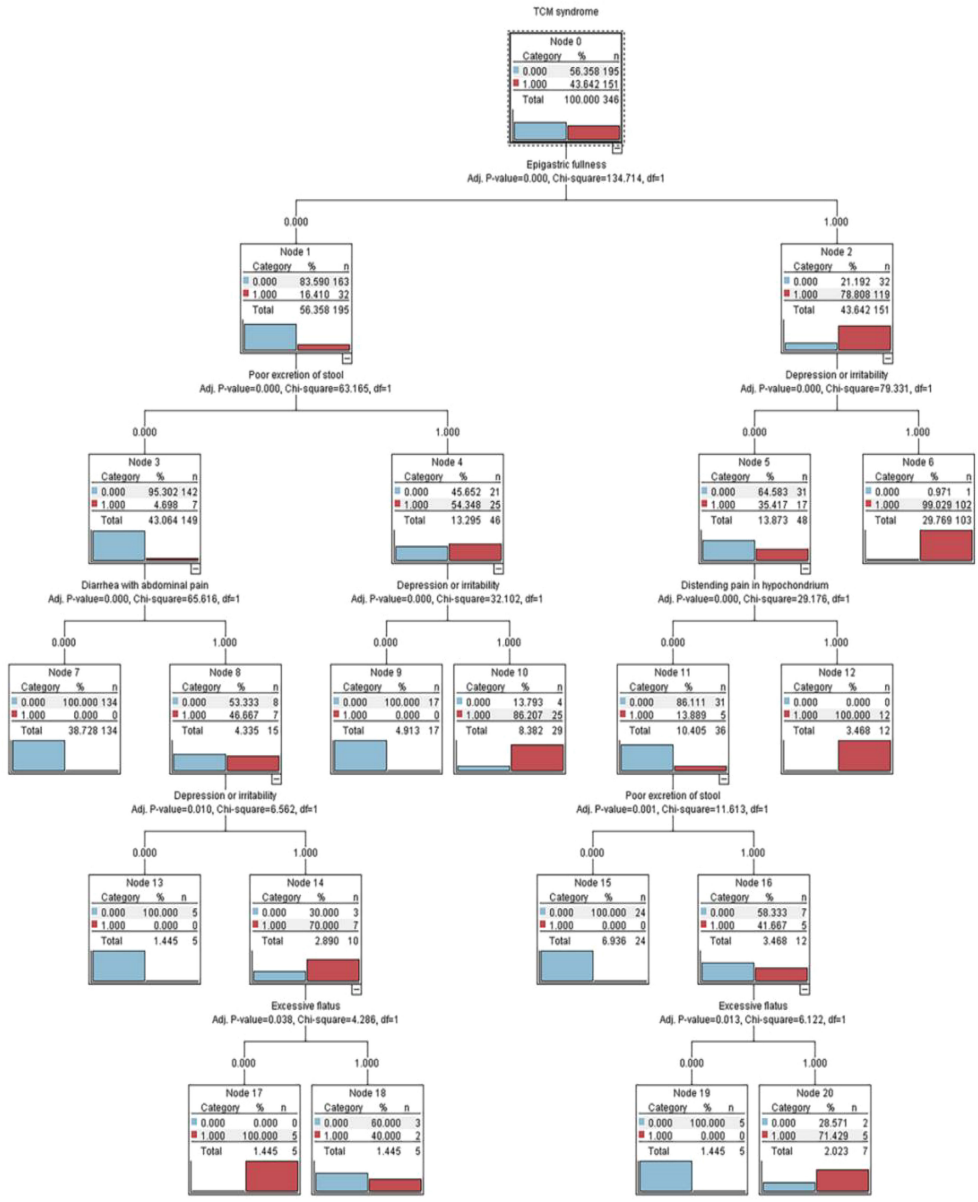
LSDS diagnostic model based on CHAID algorithm. “1” = LSDS, “0” = NLSDS. From 27 syndrome factors, six variables of epigastric fullness, depression or irritability, poor excretion of stool, distending pain in the hypochondrium, diarrhea with abdominal pain, and excessive flatus were selected to form a decision-tree model. This model, with a depth of five, 21 nodes, and 11 terminal nodes, forms the recognition route of 11 LSDS patients.

The CHAID algorithm was used to perform a decision-tree analysis of the 27 syndrome factors, and six variables were selected to form a decision-tree model (Figure 5). This model, with a depth of 5, 21 nodes, and 11 terminal nodes, forms the recognition route of 11 LSDS patients. Epigastric fullness was the first identification factor for LSDS. In the case of epigastric fullness, the next identifying factor was depression or irritability. In the absence of depression or irritability, the next discriminating factor was distending pain in the hypochondrium. In the absence of epigastric fullness, the next identifying factor was poor excretion of stool. If poor excretion of stool was present, the next distinguishing factor was depression or irritability. If poor excretion of stool does not exist, it was diarrhea with abdominal pain.

**FIGURE 6:**
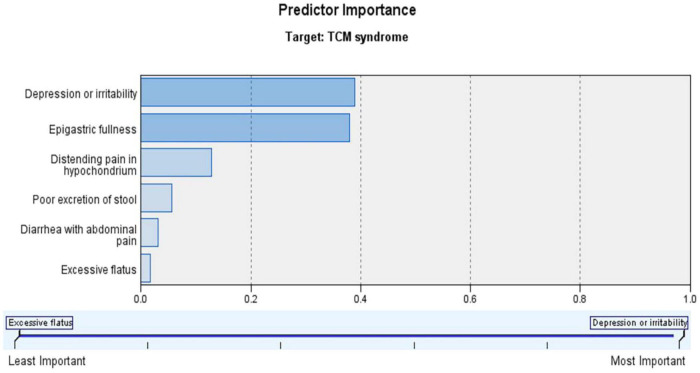
Prediction plot of variable importance based on CHAID model. The main symptoms of LSDS were ranked in order of importance. The order of symptom importance is depression or irritability, epigastric fullness, distending pain in hypochondrium, poor excretion of stool, diarrhea with abdominal pain, and excessive flatus.

The diagnostic variables of the LSDS were ranked in order of importance to identify the features important for the model’s diagnosis. Depression or irritability was the most important symptom variable of LSDS, followed by epigastric fullness and distending pain in hypochondrium. Poor excretion of stool, diarrhea with abdominal pain, and excessive flatus also played important roles in the model diagnosis (Figure 6). Based on the CHAID decision tree, a simplified diagnostic rule set has been provided in Supplementary Table S1 for potential clinical use.

## 4 Discussion

In this study, we constructed a diagnostic model for LSDS of insomnia and gastralgia based on six machine learning algorithms. The results demonstrated strong performance across all models, with the CHAID model emerging as the top performer, exhibiting the highest AUC (0.889; 95%CL 0.861–0.914). Its accuracy, sensitivity, specificity, positive predictive value, F1 score, and negative predictive value surpassed those of other models. By predicting the importance of the diagnostic variables of LSDS, we identified key factors such as depression or irritability, epigastric fullness, distending pain in the hypochondrium, poor excretion of stool, and other symptoms crucial for diagnosis. This study underscores the unique advantages of machine learning algorithms in the clinical diagnosis and treatment of TCM, facilitating the objective development of syndrome diagnosis.

TCM views an individual as an organic whole, requiring practitioners to systematically analyze symptoms, signs, and medical history through the four diagnostic methods of “looking, listening, asking, and cutting.” Unlike modern medical examinations, TCM diagnosis heavily relies on practitioners’ knowledge and subjective experience, potentially leading to varying diagnoses for the same ailment. Accurate diagnosis of TCM syndromes plays a crucial role in disease treatment, yet the complex and diverse characteristics of these syndromes make it difficult for traditional data analysis methods to comprehensively extract and analyze clinical diagnosis and treatment features (33). Therefore, the establishment of an accurate and efficient syndrome diagnosis model is conducive to the quantitative processing of diagnostic information. It is also essential for standardizing TCM diagnosis.

Machine learning has made significant strides in various medical domains, including disease diagnosis, treatment, prognosis evaluation, and new drug research, owing to its powerful data mining and processing capabilities (34, 35). For instance, Yuan et al. (36) used a machine learning algorithm to construct three types of gastric cancer diagnostic models based on tongue images and found that compared with traditional blood biomarkers, tongue images, and tongue coating microorganisms had significant advantages in the diagnosis of gastric cancer. Similarly, Liu et al. (37) employed the XGBoost algorithm to construct a diagnostic model for the TCM damp-heat mode in type 2 diabetes, while Tang et al. (38) used insomnia as an example and a machine-learning algorithm to study the clinical diagnosis and treatment rules of TCM-dominant diseases. Previous studies have shown that machine learning provides a new direction for the modernization of TCM diagnosis and treatment technologies (30).

As a prevalent decision tree algorithm, CHAID effectively summarizes the characteristics of the entire dataset. Therefore, classification predictions and rule extraction are typically employed (39). This study is the first to use a variety of machine learning algorithms to construct a diagnostic model of LSDS. Compared with traditional diagnostic models and other machine learning algorithms, CHAID enables categorical monitoring of dependent variables based on independent ones. Given the intricate, abstract, and multilevel nature of TCM syndromes, it can segment the dataset and form subsets with different syndrome characteristics. Moreover, LSDS data from different sources and sizes can be flexibly processed, and the segmentation and stopping criteria can be adjusted to prevent overfitting or underfitting (40). Additionally, CHAID can incorporate a priori knowledge of TCM evidence, such as TCM evidence elements, into model construction, thereby mining implicit laws within LSDS data, and enhancing the accuracy and credibility of the model’s diagnosis. Previous studies have underscored the pivotal role of the CHAID algorithm in disease diagnosis and prediction (41), and the results of this study also prove that the CHAID model has unique advantages in LSDS diagnosis of insomnia and gastralgia.

LSDS represents a common TCM syndrome in patients experiencing insomnia and gastralgia. According to the TCM five-element theory, the liver and spleen are mutually exclusive. The liver regulates mental and emotional states, while the spleen facilitates food digestion and absorption. If emotions and feelings are uncomfortable, liver qi is depressed and crosses over to the spleen, leading to loss of tonic function in the spleen, splenic deficiency and dampness, and an imbalance between liver and spleen, resulting in insomnia and gastralgia. With the progress of social development and competitive pressure, the incidence of mental disorders, such as anxiety and depression, is increasing every year. Studies have shown that insomnia and gastralgia are closely related to bad moods, and the presence and severity of both are significant clinical features of patients with depression and anxiety (42–44). TCM states that the physiology and psychology of the human body interact with each other and that irritability and anger injure the liver, while excessive thinking affects the spleen. Prolonged anxiety and depression can lead to the development of LSDS. However, current diagnostic criteria for LSDS in insomnia and gastralgia lack standardization.

In this study, we employed a machine-learning algorithm, utilizing the CHAID model for the first time to construct an LSDS diagnosis model. Additionally, we predicted the importance of variables in the model, highlighting depression or irritability as the most prominent symptom associated with LSDS. Other significant symptoms include epigastric fullness, distending pain in the hypochondrium, poor excretion of stool, diarrhea with abdominal pain, and increased flatulence, aligning with the LSDS theory of TCM syndrome differentiation.

From the perspective of TCM theory, the six core symptom variables identified in this study have clear pathophysiological implications. First, depression or irritability ranked as the most important symptom, reflecting the core pathogenesis of “liver qi depression.” The liver governs free flow and regulates both qi dynamics and emotional states. When liver qi is depressed, its free flow function is impaired, leading to qi stagnation and emotional disturbance, which manifests as depression or irritability. Second, epigastric fullness ranked as the second most important symptom, reflecting the pathogenic transmission of “wood overacting on earth.” The liver corresponds to wood and the spleen to earth in the five-element theory. Prolonged liver qi depression transversely invades the stomach, causing failure of stomach qi to descend and qi stagnation, which manifests as epigastric fullness. Distending pain in the hypochondrium, the third most important symptom, is a typical manifestation along the liver meridian and serves as direct evidence of liver qi depression and meridian qi stagnation. Furthermore, the intestinal manifestations—poor excretion of stool, diarrhea with abdominal pain, and excessive flatus—reflect the pathogenesis of “spleen deficiency with dampness retention.” Longstanding liver qi depression leads to failure of the liver to assist the spleen, resulting in spleen dysfunction, water-dampness retention, failure of clear qi to ascend and turbid qi to descend, which manifests as poor excretion of stool, diarrhea with abdominal pain, and excessive flatus. Together, these six symptom variables form a pathogenic evolution chain of “liver depression—spleen deficiency—dampness retention,” progressing from the liver to the spleen and from qi to dampness. This chain is highly consistent with classical TCM theory and provides an objective basis for clinical syndrome differentiation.

Previous studies have also identified depression or irritability, epigastric fullness, poor stool excretion, and diarrhea with abdominal pain as key clinical features of LSDS in patients with hepatitis B-associated liver fibrosis (45). Diarrhea with abdominal pain and depression are likewise objective indicators in the evaluation of animal models of LSDS in irritable bowel syndrome (46, 47). Thus, establishing LSDS diagnostic models for insomnia and gastralgia could offer an objective basis for the clinical differentiation of LSDS.

This study has the following advantages. Compared with previous machine learning-based TCM diagnostic studies, most existing work has focused on single diseases [e.g., insomnia (38), gastric cancer (36), and damp-heat pattern of type 2 diabetes mellitus (37)]. In contrast, this study is the first to target patients with comorbid insomnia and gastralgia for the diagnosis of LSDS, filling a gap in the field of comorbidity pattern diagnosis. First, regarding algorithm comparison. Most previous studies employed a single algorithm, whereas this study systematically compared the diagnostic performance of six machine learning algorithms on the same dataset. The results showed that the CHAID model achieved the best performance, demonstrating significant advantages in classification prediction and rule extraction. Second, regarding model interpretability. The CHAID decision tree model not only exhibited superior diagnostic performance but also provided a clear decision pathway and variable importance ranking. This study identified six major symptom variables for LSDS, offering clinicians an intuitive and actionable diagnostic basis. Third, regarding clinical value. This diagnostic model can promote the objectification and standardization of LSDS diagnosis, assist young and primary care physicians in clinical assessment, and holds potential for application in TCM training and internet-based diagnostic platforms.

This study has several limitations. First, the data were collected from only two tertiary hospitals without independent external validation; therefore, the generalizability of the model to primary care and real-world populations requires further confirmation. Second, the diagnostic gold standard was based on TCM expert judgment, which is inherently subjective. Although we implemented standardized training and group consensus to mitigate this bias, inter-rater reliability was not prospectively assessed. Of note, this model is not intended to replace expert TCM diagnosis but may serve as an auxiliary tool to support syndrome standardization and practitioner training. Future multi-center external validation and integration with objective indicators (e.g., endoscopy, biomarkers) are needed to further evaluate its clinical value.

Misclassification of LSDS carries potential clinical risks. Overdiagnosis may lead to unnecessary treatment with liver-spleen harmonizing herbs, exposing patients to potential adverse effects, increasing healthcare costs, and delaying appropriate alternative interventions. Underdiagnosis may result in missed opportunities for effective TCM treatment, potentially prolonging patient discomfort and reducing quality of life. These considerations underscore the importance of prudent model application in clinical practice.

## 5 Conclusion

In this study, we successfully established diagnostic models for Liver-Spleen Disharmony Syndrome (LSDS) in patients with insomnia and gastralgia using six machine learning algorithms based on TCM four-diagnosis information. Among the six algorithms, the CHAID decision tree model demonstrated the best performance, exhibiting not only good diagnostic performance but also favorable interpretability. This model provides a novel and objective auxiliary tool for the clinical identification of LSDS and contributes to advancing the standardization and quantification of TCM syndrome diagnosis. The model is currently at the developmental stage, and further validation and optimization in independent, multi-center external cohorts are needed before it can be applied clinically. Ultimately, this model holds the potential to provide clinicians, especially those in primary care settings, with a convenient and accurate tool to support diagnostic decision-making.

## Data Availability

The raw data supporting the conclusions of this article will be made available by the authors, without undue reservation.
